# The many greenhouse gas footprints of green hydrogen†

**DOI:** 10.1039/d2se00444e

**Published:** 2022-08-24

**Authors:** Kiane de Kleijne, Heleen de Coninck, Rosalie van Zelm, Mark A. J. Huijbregts, Steef V. Hanssen

**Affiliations:** Department of Environmental Science, Radboud Institute for Biological and Environmental Sciences, Radboud University P.O. Box 9010, 6500 GL Nijmegen The Netherlands Kiane.deKleijne@ru.nl; Technology, Innovation and Society Group, Department of Industrial Engineering and Innovation Sciences, Eindhoven University of Technology P.O. Box 513 5600 MB Eindhoven The Netherlands

## Abstract

Green hydrogen could contribute to climate change mitigation, but its greenhouse gas footprint varies with electricity source and allocation choices. Using life-cycle assessment we conclude that if electricity comes from additional renewable capacity, green hydrogen outperforms fossil-based hydrogen. In the short run, alternative uses of renewable electricity likely achieve greater emission reductions.

## Abstract 2

This communication shows how green hydrogen's climate footprint and performance against alternatives depends on electricity source and accounting choices.

## Graphical abstract

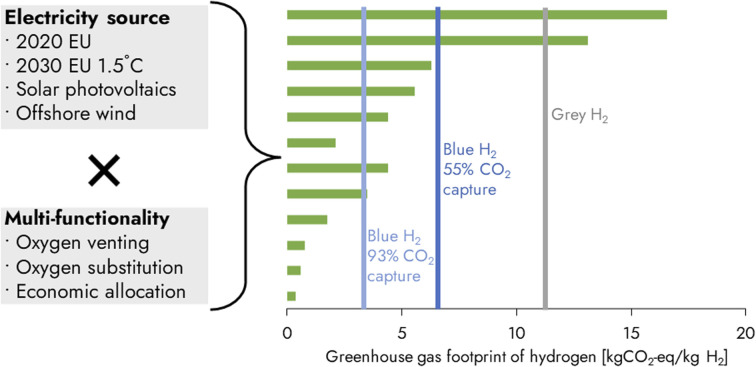

Hydrogen is widely considered both a promising industrial feedstock and a potentially important future energy carrier in the context of decarbonisation.^1–3^ The current global hydrogen demand of 75 million tonnes per year is predominantly met with so-called ‘grey’ hydrogen, produced from natural gas.^4^ Alternative lower-emission hydrogen production methods have led to a colourful palette of hydrogen types, including ‘blue’ hydrogen produced from natural gas combined with carbon capture and storage, and ‘green’ hydrogen produced *via* water electrolysis using renewable electricity. In the IEA Sustainable Development Scenario,^4^ these methods of hydrogen production are projected to scale up towards 200 million tonnes of blue and 300 million tonnes of green hydrogen by 2070.^4^ Hydrogen can also be produced from biomass,^5^ though this route is currently less technologically mature, depends on the availability of biomass against competing uses, and is more costly compared to green and blue hydrogen, which are therefore the focus for low-emission hydrogen production.^1^

The estimated climate impacts of the various hydrogen production routes vary widely in the life-cycle assessment (LCA) literature, which may confuse the best course of action on low-emission hydrogen for policymakers, investors and consumers. Blue hydrogen was recently found to reduce greenhouse gas (GHG) emissions compared to grey hydrogen by 5–36%,^6^ while a different set of assumptions for upstream methane leakage and carbon capture rates leads to a reduction of 26–75% compared to grey hydrogen.^7^ The source of electricity causes large variations in the GHG footprint of electrolytic hydrogen^1,3,7–10^ with up to 200% difference (*i.e.*, the absolute difference divided by the average), as does the ‘multi-functionality’ question of how to allocate GHG emissions between hydrogen and co-produced oxygen (158% difference^11^). GHG footprints of green hydrogen specifically, vary as a result of the use of different renewable electricity sources (wind or solar photovoltaics): 102–120% difference,^9^ different electrolysis technologies (alkaline electrolysis or polymer electrolyte membrane electrolysis): 16–40% difference,^9^ and various assumptions on future improvements (increased efficiency and lifespan): 18% difference.^8^ The wide range in GHG footprints of green hydrogen warrants additional understanding of how these footprints are assessed, how they have come to diverge and what is required to lower them.

Of specific concern for green hydrogen is the principle of additionality,^12^ which refers to producing green hydrogen using only newly installed, additional, renewable electricity capacity that matches the increased demand from electrolysers (thereby preventing additional fossil-based electricity generation). The relevance of additionality is illustrated by the European Commission's 2020 hydrogen strategy that foresees a green hydrogen production of 10 million tonnes by 2030,^2^ which would require 140% of the 394 TW h of electricity generated by all wind turbines in the European Union (EU) in 2020 (ref. 13) at an electricity requirement of 55 kW h kg^−1^ of hydrogen.^10^ Realising these green hydrogen targets implies that renewable electricity generation needs to be increased or diverted from other uses.

Here, we evaluate how the GHG footprint of green hydrogen depends on three choices: (i) the (future) electricity source; (ii) the multi-functionality approach; and (iii) the grey or blue hydrogen benchmark against which the emissions are compared (Fig. 1). We focus on polymer electrolyte membrane (PEM) electrolysers as the new generation, which is more efficient^9^ and less material-intensive^10^ compared to the more mature alkaline electrolysers. To illustrate how the combinations of different choices would result in different policy conclusions, we calculate the GHG footprint of electrolytic hydrogen produced in the EU for all combinations of these choices, based on the LCA ISO 14044 guideline. Furthermore, we assess how the use of additional renewable electricity for hydrogen compares to alternative uses of this electricity in terms of climate change benefits. Last, we suggest a way forward to comprehensively inform decisions of consumers, investors and policymakers based on ranges in GHG footprinting studies.

**Fig. 1:**
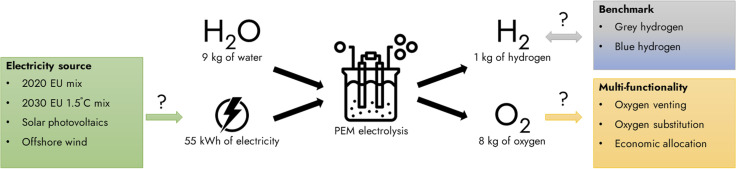
Production scheme and choices for the electricity source, multi-functionality approach (*i.e.*, method of accounting for co-products) and benchmark for electrolytic hydrogen serving as the basis for our LCA. 1 kg of hydrogen output is associated with 8 kg of oxygen output, requires 55 kW h of electricity including compression, 9 kg of ultrapure water and infrastructure for the polymer electrolyte membrane (PEM) electrolyser stack and balance of plant.^10^

## Electricity source

Green hydrogen is by definition produced through electrolysis using renewable electricity. Still, green hydrogen's GHG footprints vary depending on the exact renewable electricity source and on whether or not additional renewable capacity is used for hydrogen production. With regard to the latter, we argue that since electrolysers cannot be considered to solely use the green component in the grid mix, they essentially run on the average grid mix unless additionality is guaranteed.

**Fig. 2:**
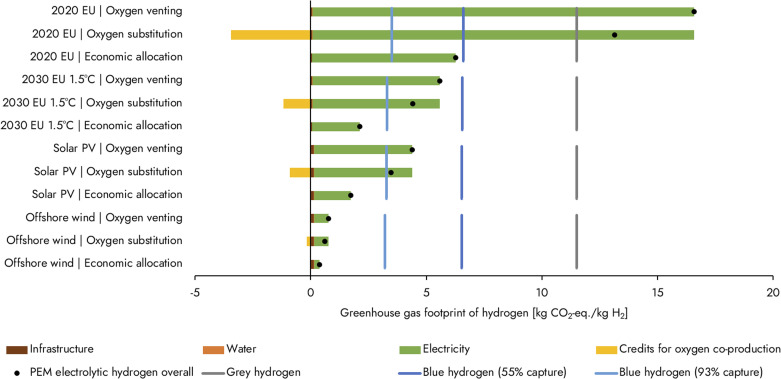
The greenhouse gas footprint of PEM electrolytic hydrogen for different electricity sources and multi-functionality approaches in kg CO_2_-equivalents (kg_CO_2_-eq._) per kg H_2_. The benchmarks grey and blue hydrogen (the latter with a CO_2_ capture rate of 55% or 93%) are based on Bauer *et al.*^7^ and were harmonised for each electricity source. The 2020 EU electricity mix and the 2030 mix for a 1.5 °C-consistent emissions pathway are based on the integrated assessment model REMIND;^25^ solar PV located in Europe was based on Bosmans *et al.*;^14^ offshore wind was based on Bonou *et al.*^26^ Infrastructure emissions are based on 3000 full-load hours for the purely renewable sources while on 8000 full-load hours for the grid mixes. LCA inputs were based on Bareiß *et al.*^10^ and background life-cycle inventory data on the Ecoinvent database version v3.7.1, using allocation at point of substitution. The ReCiPe2016 method (H) v1.05 was used at midpoint level to quantify the greenhouse gas footprints. Details on how we applied different methods to deal with multi-functionality can be found in the ESI.† The contribution of oxygen is shown as having a negative greenhouse gas footprint because it is a prevented emission; it does not signify carbon dioxide removal.

Fig. 2 shows the GHG footprint of green hydrogen produced with different renewables (wind or solar) and current and future average grid electricity, calculated based on the life-cycle inventory in Bareiß *et al.*^10^ (see ESI† for details). Hydrogen powered by offshore wind (GHG footprints of 0.4–0.8 kg_CO_2_-eq._ kg_H_2__^−1^) results in a twentyfold (93–97%) reduction in GHG footprint compared to grey hydrogen and a reduction of 76–94% compared to blue hydrogen. These results are comparable to the value of 0.7 kg_CO_2_-eq._ kg_H_2__^−1^ for wind-based hydrogen (with oxygen venting) reported by Mac Dowell *et al.*,^3^ who included additional GHG emissions for hydrogen storage.

Using solar PV for hydrogen production leads to a GHG footprint of 1.7–4.4 kg_CO_2_-eq._ kg_H_2__^−1^ and equates to a 62–85% reduction compared to grey hydrogen and is in the same range as blue hydrogen (34% increase to 73% reduction in GHG emissions compared to blue hydrogen; see Fig. 2). The GHG footprint of hydrogen produced with solar PV electricity is approximately five times larger compared to offshore wind-based hydrogen, illustrating the large differences across different renewable electricity sources. The GHG intensity of renewable electricity is also location-dependent,^14^ which in turn affects hydrogen produced with it. Still, in absolute terms GHG footprints of green hydrogen are clearly low if based on renewables, and may further decrease in the future if higher electrolysis efficiencies can be obtained.^8,9^

Using the 2020 EU grid mix, for the case of non-additionality, electrolytic hydrogen has a GHG footprint of 6.3–16.6 kg_CO_2_-eq._ kg_H_2__^−1^, which is in most cases higher than grey hydrogen (Fig. 2). A cleaner 2030 grid mix (compatible with the EU targets for limiting warming to 1.5 °C), results in a lower, but still sizable GHG footprint (2.1–5.6 kg_CO_2_-eq._ kg_H_2__^−1^) in the range of blue hydrogen.

## Alternative uses of new renewable electricity

**Fig. 3:**
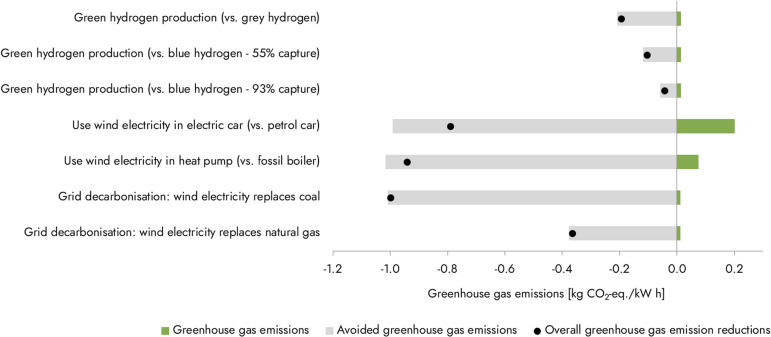
Greenhouse gas emissions and avoided emissions for different ways of using 1 kW h produced from newly built offshore wind capacity in kg CO_2_-equivalents (kg_CO2-eq._) per kW h. The values for green and blue hydrogen are the same as in Fig. 2, under the assumption that oxygen is vented. Emissions and avoided emissions for electric vehicles and heat pumps in the EU are calculated based on Knobloch *et al.*,^17^ and for grid decarbonisation based on Hertwich *et al.*^18^ Details on how we calculated the greenhouse gas emissions and avoided emissions can be found in the ESI.† Negative values are shown for avoided emissions and emission reductions, and do not signify carbon dioxide removal.

While green hydrogen has low GHG footprints when based on additional renewables, the question remains whether green hydrogen production is the best way to use renewable electricity sources. From a climate perspective, it has been argued that priority should be given to technologies with the highest emission reduction potential per kW h of renewable electricity.^15,16^Fig. 3 shows the emission reduction potentials for the use of 1 kW h of newly built offshore wind power for green hydrogen production replacing either grey or blue hydrogen, for electric cars replacing petrol cars,^17^ heat pumps replacing fossil boilers,^17^ or directly decarbonising the existing grid mix by replacing coal or natural gas electricity.^18^ Here we see that green hydrogen production replacing grey hydrogen production results in approximately 2–5 times smaller emission reduction per kW h of renewable electricity compared to the competing uses. In the short run, while the grid can still be further decarbonised and electrification of heat and transport is still in progress, these applications of renewable electricity may take priority over green hydrogen production, if climate benefits are to be maximised. Only when (local) renewable electricity demand for the alternatives has been met, would green hydrogen production be effective in contributing to emission reductions. An important advantage of green hydrogen production from fluctuating renewable electricity compared to the alternatives is that it can be produced, when combined with hydrogen storage, when demand for renewable electricity elsewhere is low. This leaves opportunities for hydrogen production in areas with large renewable energy potentials, including renewable capacity without grid connection.^16^

## Multi-functionality

Oxygen that is co-produced in water electrolysis is typically vented to the air,^10^ meaning that all GHG emissions are allocated to the hydrogen produced. Alternatively, oxygen can be further purified for use in a subsequent process. Already in 2005, options to utilise the co-produced oxygen were explored as a way to bring down the relative economic costs of electrolytic hydrogen,^19^ and recently this has attracted renewed interest.^20^ Analogously, the valorisation of co-produced oxygen has been included in footprinting studies (*e.g.*, ref. 11 and 21) in which part of the emissions of the hydrogen production process are allocated to the utilised oxygen. The ISO standards for LCA (ISO 14044) provide a hierarchy of methods to deal with this division, but leave freedom for the practitioner to choose an approach. When using a ‘system expansion *via* substitution’ approach it is assumed that co-produced oxygen replaces conventional oxygen production *via* air separation elsewhere (details can be found in the ESI†). As shown in Fig. 2, this results in a considerably lower net GHG footprint for hydrogen compared to when oxygen is vented.

A second option to deal with multi-functionality is allocation of emissions based on economic value. Using recent market prices for hydrogen and oxygen to represent their economic value, the GHG footprint of electrolytic hydrogen under economic allocation is approximately halved compared to substitution (Fig. 2). This leads to different conclusions on whether electrolytic hydrogen reduces emissions compared to benchmark blue or grey hydrogen.

When considering electrolysis at scale, large quantities of co-produced oxygen could lead to a market saturated for oxygen. Comparing the estimated European oxygen market of 17 million tonnes per year^22^ to the 80 million tonnes of oxygen that would be co-produced with the 10 million tonnes green hydrogen target for 2030,^2^ it is clear that there would likely be an oxygen surplus, even if the market for oxygen grows. This would make continued substitution of conventional oxygen production unrealistic and would diminish the share of emissions that can be economically allocated to oxygen. Beyond oxygen market saturation, virtually all emissions can be assigned to hydrogen, leaving oxygen venting as both a conservative but also realistic default option.

## Benchmark

The emission reduction potential of deploying electrolytic hydrogen depends on the benchmark against which it is assessed. Electrolytic hydrogen could first replace existing uses of grey hydrogen *e.g.* as feedstock in the chemical industry, in fertiliser production, as reducing agent in the steel industry, or as transport fuel.^1^ However, the benchmark could shift over time: grey hydrogen production facilities can be retrofitted to include carbon capture and storage (CCS) leading to blue hydrogen production, either capturing CO_2_ from the steam methane reforming process only (55% capture rate), or also from the flue gas (93% capture rate). The GHG intensity of electricity used in green hydrogen production has to be sufficiently low to outperform this new benchmark of blue hydrogen; the cut-off is approximately 58 g_CO_2_-eq._ kW^−1^ h^−1^ (assuming oxygen is vented for green hydrogen and a 93% carbon capture rate for blue hydrogen), which is lower than the median value for solar PV electricity in Europe. The benchmark for (green) hydrogen application in industry and transport, *i.e.*, what are realistically substituted products, could also shift over time, warranting careful interpretation of green hydrogen's long term emission reduction potential. A potential additional concern for all hydrogen pathways is leakage of hydrogen, which may increase its GHG footprint; research on this has only recently started.^23^

In the context of green hydrogen's comparison against grey hydrogen, there is a technical, but critical concern that the often used Ecoinvent LCA database erroneously reports a fossil hydrogen GHG footprint that is five times lower (2.2 kg_CO_2_-eq._ kg_H_2__^−1^) compared to the well-established footprint in literature of around 11.5 kg_CO_2_-eq._ kg_H_2__^−1^ for grey hydrogen^7,10^ (details are provided in the ESI;† the misrepresentation has been communicated to the database developers and a new process for grey hydrogen production will be added in the next version). The use of this Ecoinvent value in LCA studies has led to incorrectly low GHG footprints (*e.g.*, ref. 21 and 24).

## Implications

Our results illustrate that, depending on the choices in the calculation of the GHG footprint of green hydrogen (electricity source, multi-functionality and benchmark), green hydrogen production in the EU can achieve no, a small, or a large emission reduction. This dependency extends to many other products that are electricity-intensive or involve co-products, including the electrification of transport and the production of metals, as their footprints depend on the same choices. These accounting choices also interact: with a large electricity GHG footprint like the 2020 EU electricity mix, the allocation choice has a large effect on whether or not electrolytic hydrogen reduces emissions compared to the benchmark. Conversely, for a small electricity GHG footprint like for wind power, no matter which multi-functionality approach is used, the GHG footprint is lower than of the benchmarks.

To indicate how much the choice of electricity source contributes to the calculated GHG footprint of green hydrogen, and how much the multi-functionality approach, we performed a variance decomposition analysis. The results show that the majority of variation in the GHG footprint is attributable to the GHG intensity of the electricity mix (92% of variance explained); the remaining 8% of the variation is attributable to the multi-functionality approach. From this we conclude that policies focusing on the GHG footprint of electricity used in electrolysis is most important in green hydrogen production.

Although green hydrogen could achieve the lowest GHG footprints in the long run, in the transition period until low-GHG electricity sources are readily available, deploying blue hydrogen may achieve higher emission reductions than green hydrogen. In addition, blue hydrogen may have lower environmental impacts than green, wind-based hydrogen when looking at impact categories beyond climate change, for example regarding mineral resource scarcity and freshwater ecotoxicity.^3^ This could indeed call for a “twin track” approach,^3^ in which blue hydrogen and green hydrogen are deployed and scaled up simultaneously, taking into account regional preferences, environmental impacts, and economic and political interests.

We have shown here that the GHG footprint of green hydrogen strongly varies depending on a small number of choices. While footprints can inform and guide policymakers, investors and consumers, it is important to understand the context for which footprints are derived. Footprints ought to be viewed not just as a single number, but it needs to be considered how they change under different scenarios, choices and assumptions, over time, and how they compare to alternative options. This could steer actions that facilitate change in the desirable direction. The case of green hydrogen illustrates this: it is a no-regret for policymakers to reinforce decarbonisation of the electricity mix, to ensure that if electricity is used for hydrogen production, it has a low GHG footprint, and to ensure that if renewable power capacity is used for hydrogen production, it is not diverted from other uses that can achieve higher emission reductions. Only then, hydrogen is really ‘green’ and can fulfil its environmental promise as energy carrier and industrial feedstock.

## Conflicts of interest

There are no conflicts to declare.

## Supplementary Material

SE-006-D2SE00444E-s001
