# Supplementary material for: The many greenhouse gas footprints of green hydrogen
Source: Sustain Energy Fuels. 2022 Aug 24;6(19):4383–7. doi: 10.1039/d2se00444e (PMC9514062; doi:10.1039/d2se00444e)
Supplement: SE-006-D2SE00444E-s001 [file SE-006-D2SE00444E-s001.pdf]

Electronic Supplementary Material (ESI) for Sustainable Energy & Fuels.
This journal is © The Royal Society of Chemistry 2022

Supplementary Information

The many greenhouse gas footprints of green hydrogen

Kiane de Kleijne*, Heleen de Coninck, Rosalie van Zelm, Mark A.J. Huijbregts and Steef V. Hanssen

Correspondence: Kiane.deKleijne@ru.nl

Sections

1 | Methods for greenhouse gas footprint calculation hydrogen *(Figure 2)*

2 | Methods for the comparison of green hydrogen against alternative uses of renewable electricity *(Figure 3)*

3 | Supplementary results: comparison of green hydrogen against alternative uses of solar PV electricity

4 | Detailed description of the misrepresentation of fossil hydrogen greenhouse gas footprints in Ecoinvent v3.7.1

1. Methods for greenhouse gas footprint calculation hydrogen

We calculated the greenhouse gas (GHG) footprint of polymer electrolyte membrane (PEM) electrolytic hydrogen for different electricity sources and multi-functionality approaches in kg CO_2-equivalents (kgCO_2-eq) per kg H_2 (Figure 2). The inputs for the life-cycle assessment (LCA), using a functional unit of 1 kg of hydrogen produced, can be found in Table S1. These are based on Bareiß et al.[1] and background life-cycle inventory data on the Ecoinvent database version v3.7.1, using allocation at point of substitution. The ReCiPe2016 method (H) v1.05 was used at midpoint level to quantify the GHG footprints. The four electricity sources used to calculate the GHG footprint are detailed in Table S2. Details on how we applied different methods to deal with multi-functionality can be found in Table S3. The GHG footprints of the benchmarks grey and blue hydrogen are less dependent on the GHG intensity of the electricity source because they mainly require natural gas as input. Bauer et al.[2] showed the contribution of electricity to the GHG footprint of grey and blue hydrogen in a contribution analysis. Based on this, we harmonised the electricity used in the electrolysis process and the grey/blue hydrogen production processes, see Table S4.

Table S1 | Life-cycle inventory for PEM electrolytic hydrogen based on Bareiß et al.[1].

**Inputs**	
Water	9.0 kg
Electricity	55 kWh based on an efficiency of 60% lower heating value. Compression is included.
Infrastructure (polymer electrolyte membrane electrolyser stack and the balance of plant)	We directly used the global warming impact of 0.132 kgCO_2-eq per kg H_2 from Bareiß et al.[1] based on 3000 full-load hours in the case of using renewable electricity. For the grid mix cases, we scaled this value to be in line with 8000 full-load hours.
**Outputs**	
Hydrogen	1.0 kg
Oxygen	8.0 kg

Table S2 | GHG intensity of electricity sources.

**Electricity source**	**Notes**
2020 EU electricity grid	0.30 kgCO_2-eq/kWh based on the 2020 GHG footprints in Layritz et al.[3], which is in line with the latest EUROSTAT data.
2030 1.5˚C-compatible EU electricity grid	0.10 kgCO_2-eq/kWh[3].
Solar photovoltaics (PV)	0.077 kgCO_2-eq/kWh, the median GHG footprint of current and planned solar PV facilities in Europe[4].
Offshore wind	0.011 kgCO_2-eq/kWh including end of life[5].

Table S3 | Multi-functionality approaches.

Multi-functionality approach	Notes
Venting of oxygen	The full climate impact is assigned to hydrogen.
Substitution of oxygen	To determine the GHG footprint of 1.0 kg hydrogen production, the GHG footprint of 8.0 kg of conventional oxygen production (via cryogenic air separation) is subtracted from the footprint of the overall production process. We used the Ecoinvent v3.7.1 process 'Market for oxygen, liquid {RER}' and adapted this process to ensure harmonisation of the electricity used in electrolysis and the substituted oxygen production process. We replaced the electricity source for the required 1.42 kWh/kg O_2 in the Ecoinvent process by the four electricity sources specified in Table S2. This approach avoids that benefits of substitution would be inflated by a replaced process that runs on more GHG-intensive electricity.
Economic allocation	We used the factors in Bargiacchi et al.[6] based on the 2020 prices of hydrogen (1.21 USD/kg) and oxygen (0.25 USD/kg). This led to assigning 37.7% of the impacts to hydrogen and 62.3% to oxygen.

Table S4 | Benchmarks.

Benchmark	Notes
Grey hydrogen	For a CH_4 emission rate of 1.5%, GWP100: 11.5 kgCO_2-eq/kg H_2. Essentially no electricity used[2]. This value is therefore independent of electricity source.
Blue hydrogen (55% capture)	For a CH_4 emission rate of 1.5%, GWP100 and a 55% CO_2 capture rate Bauer et al.[2] found a GHG footprint of 6.63 kgCO_2-eq/kg H_2, of which 0.11 kgCO_2-eq/kg H_2 is due to electricity use[2]. This equals 0.28 kWh/kg H_2 based on the indicated ENTSO-E GHG intensity (0.39 kgCO_2-eq/kWh), which we replaced by the four electricity sources specified in Table S2 to find the harmonised blue hydrogen (55% capture) footprints.
Blue hydrogen (93% capture)	For a CH_4 emission rate of 1.5%, GWP100 and a 93% CO_2 capture rate Bauer et al.[2] found a GHG footprint of 3.61 kgCO_2-eq/kg H_2, of which 0.41 kgCO_2-eq/kg H_2 due to electricity use[2]. This equals 1.04 kWh/kg H_2 based on the indicated ENTSO-E GHG intensity (0.39 kgCO_2-eq/kWh), which we replaced by the four electricity sources specified in Table S2 to find the harmonised blue hydrogen (93% capture) footprints.

Table S5 | Values underlying Figure 2.

Electrolytic hydrogen							Grey hydrogen	Blue hydrogen (55% capture)	Blue hydrogen (93% capture)
**Electricity source**	**Multi-functionality approach**	**Infrastructure ($kgCO_2$-eq/kg H_2)**	**Water ($kgCO_2$-eq/kg H_2)**	**Electricity ($kgCO_2$-eq/kg H_2)**	**Credits for oxygen co-production ($kgCO_2$-eq/kg H_2)**	**Overall GHG footprint ($kgCO_2$-eq/kg H_2)**	**GHG footprint ($kgCO_2$-eq/kg H_2)**	**GHG footprint ($kgCO_2$-eq/kg H_2)**	**GHG footprint ($kgCO_2$-eq/kg H_2)**
2020 EU	Venting of oxygen	0.05	0.03	16.50	0.00	16.57	11.5	6.60	3.51
2020 EU	Substitution of oxygen	0.05	0.03	16.50	-3.44	13.13	11.5	6.60	3.51
2020 EU	Economic allocation	0.05	0.01	6.22	0.00	6.28	11.5	6.60	3.51
2030 1.5˚C EU	Venting of oxygen	0.05	0.03	5.50	0.00	5.57	11.5	6.55	3.30
2030 1.5˚C EU	Substitution of oxygen	0.05	0.03	5.50	-1.17	4.40	11.5	6.55	3.30
2030 1.5˚C EU	Economic allocation	0.05	0.01	2.07	0.00	2.13	11.5	6.55	3.30
Solar PV	Venting of oxygen	0.13	0.03	4.24	0.00	4.39	11.5	6.54	3.28
Solar PV	Substitution of oxygen	0.13	0.03	4.24	-0.91	3.48	11.5	6.54	3.28
Solar PV	Economic allocation	0.13	0.01	1.60	0.00	1.74	11.5	6.54	3.28
Offshore wind	Venting of oxygen	0.13	0.03	0.61	0.00	0.76	11.5	6.52	3.21
Offshore wind	Substitution of oxygen	0.13	0.03	0.61	-0.16	0.60	11.5	6.52	3.21
Offshore wind	Economic allocation	0.13	0.01	0.23	0.00	0.37	11.5	6.52	3.21

2. Methods for the comparison of green hydrogen against alternative uses of renewable electricity

We calculated the GHG emissions or savings for different ways of using 1 kWh produced from newly built offshore wind capacity (Figure 3 and Table S6) and from newly built solar PV in Europe (Figure S1 and Table S7): for green hydrogen replacing grey hydrogen or blue hydrogen (55% or 93% CO_2 capture rates), for electric cars replacing petrol cars and for grid decarbonisation, replacing coal or natural gas electricity. We expressed the GHG footprints of using 1 kWh of additional renewable electricity in $kgCO_2$-equivalents per kWh of electricity. We arrived at these values by dividing the GHG footprints per original functional unit by the electricity used in kWh per functional unit, for example 55 kWh per kg of H_2 in the case of green hydrogen. For green hydrogen it is assumed that oxygen is vented, and the values are based on blue and grey hydrogen are from Table S5. Emissions and emissions savings for electric vehicles and heat pumps in the EU are calculated based on Knobloch et al.[7], where we took a weighted average of the emissions associated with electric vehicles and heat pumps for the 2022 EU-27 countries, based on absolute vehicle kilometres in each country and heat demand in each country, respectively. For grid decarbonisation we based on Hertwich et al.[8].

The *greenhouse gas emissions* in Table S6 and Table S7 are the emissions associated with the use of the electricity (e.g., green hydrogen), and the *avoided greenhouse gas emissions* are those that are avoided (e.g., avoiding grey hydrogen production) and therefore include a negative sign. The *overall greenhouse gas emission reductions* are the sum of the two.

Table S6 | Values underlying Figure 3.

	Greenhouse gas emissions ($kgCO_2$-eq/kWh)	Avoided greenhouse gas emissions ($kgCO_2$-eq/kWh)	Overall greenhouse gas emission reductions ($kgCO_2$-eq/kWh)
Green hydrogen production (vs. grey hydrogen)	0.01	-0.21	-0.20
Green hydrogen production (vs. blue hydrogen - 55% capture)	0.01	-0.12	-0.10
Green hydrogen production (vs. blue hydrogen - 93% capture)	0.01	-0.06	-0.04
Use wind electricity in electric car (vs. petrol car)	0.20	-0.99	-0.79
Use wind electricity in heat pump (vs. fossil boiler)	0.08	-1.02	-0.94
Grid decarbonisation: wind electricity replaces coal	0.01	-1.01	-1.00
Grid decarbonisation: wind electricity replaces natural gas	0.01	-0.38	-0.37

Table S7 | Values underlying Figure S1.

	Greenhouse gas emissions ($kgCO_2$-eq/kWh)	Avoided greenhouse gas emissions($kgCO_2$-eq/kWh)	Overall greenhouse gas emission reductions ($kgCO_2$-eq/kWh)
Green hydrogen production (vs. grey hydrogen)	0.08	-0.21	-0.13
Green hydrogen production (vs. blue hydrogen - 55% capture)	0.08	-0.12	-0.04
Green hydrogen production (vs. blue hydrogen - 93% capture)	0.08	-0.06	0.02
Use solar PV electricity in electric car (vs. petrol car)	0.27	-0.99	-0.72
Use solar PV electricity in heat pump (vs. fossil boiler)	0.14	-1.02	-0.88
Grid decarbonisation: solar PV electricity replaces coal	0.08	-0.86	-0.79
Grid decarbonisation: solar PV electricity replaces natural gas	0.08	-0.51	-0.43

3. Supplementary results: comparison of green hydrogen against alternative uses of solar PV electricity

In addition to comparing different uses of additional offshore wind electricity (Figure 3), in Figure S1 we show how the use of additional solar PV electricity for green hydrogen compares to alternative uses. Since solar PV has a greater GHG intensity per kWh than offshore wind electricity, the absolute numbers change compared to Figure 3 but the conclusion stays the same. Using additional solar PV electricity in Europe for electric vehicles, heat pumps or grid decarbonisation achieves greater emission reductions than using it for green hydrogen. In addition, using this electricity for green hydrogen would increase emissions compared to blue hydrogen when the CO_2 capture rate is 93%.

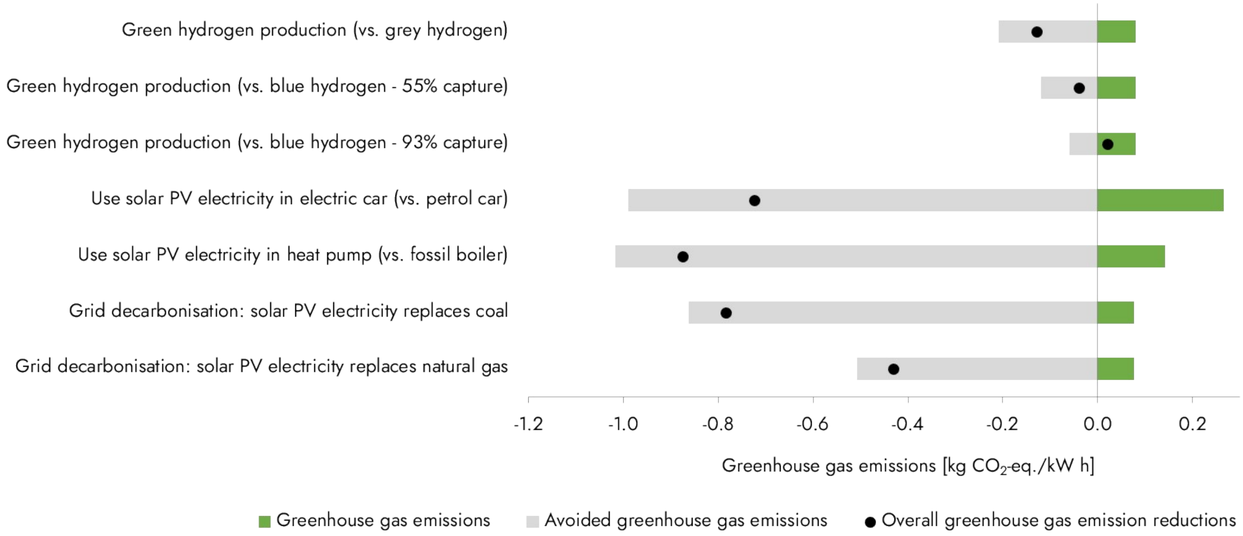

Figure S1 | Greenhouse gas emissions and avoided emissions for different ways of using 1 kW h produced from newly built solar PV capacity in kg CO_2-equivalents (kgCO_2-eq) per kW h. The values for green and blue hydrogen are the same as in Figure 2, under the assumption that oxygen is vented. Emissions and avoided emissions for electric vehicles and heat pumps in the EU are calculated based on Knobloch et al.[7], and for grid decarbonisation based on Hertwich et al.[8]. Negative values are shown for emission reductions and do not signify carbon dioxide removal.

4. Detailed description of the misrepresentation of fossil hydrogen greenhouse gas footprints in Ecoinvent v3.7.1

The GHG footprint of the average European hydrogen production process in the Ecoinvent v3.7.1 database (market for hydrogen, liquid {RER}) is reported as 2.2 kgCO_2-eq/kg H_2, i.e. five times lower than the well-established GHG footprint of conventional grey hydrogen of around 11.5 kgCO_2-eq/kg H_2[1,2]. This inconsistency is a result of the assumption in the Ecoinvent database that 96.7% of the average European production process is covered by hydrogen production during cracking of naphtha in the plastics industry[9]. Naphtha cracking yields, besides the main products ethylene and propylene, 0.01 kg H_2 by-product per kg naphtha[10]. However, this process' contribution to the actual hydrogen market is negligible. This value is therefore a misrepresentation of a conventional hydrogen production process.

Supplemental references

1 K. Bareiß, C. de la Rua, M. Möckl and T. Hamacher, *Appl. Energy*, 2019, **237**, 862–872.

2 C. Bauer, K. Treyer, C. Antonini, J. Bergerson, M. Gazzani, E. Gencer, J. Gibbins, M. Mazzotti, S. T. McCoy, R. McKenna, R. Pietzcker, A. P. Ravikumar, M. C. Romano, F. Ueckerdt, J. Vente and M. van der Spek, *Sustain. Energy Fuels*, 2022, **6**, 66–75.

3 L. S. Layritz, I. Dolganova, M. Finkbeiner, G. Luderer, A. T. Penteado, F. Ueckerdt and J.-U. Repke, *Appl. Energy*, 2021, **296**, 117049.

4 J. H. C. Bosmans, L. C. Dammeier and M. A. J. Huijbregts, *Environ. Res. Lett.*, 2021, **16**, 094056.

5 A. Bonou, A. Laurent and S. I. Olsen, *Appl. Energy*, 2016, **180**, 327–337.

6 E. Bargiacchi, N. Thonemann, J. Geldermann, M. Antonelli and U. Desideri, *Energies*, 2020, **13**, 4579.

7 F. Knobloch, S. V Hanssen, A. Lam, H. Pollitt, P. Salas, U. Chewpreecha, M. A. J. Huijbregts and J.-F. Mercure, *Nat. Sustain.*, 2020, **3**, 437–447.

8 E. G. Hertwich, T. Gibon, E. A. Bouman, A. Arvesen, S. Suh, G. A. Heath, J. D. Bergesen, A. Ramirez, M. I. Vega and L. Shi, *Proc. Natl. Acad. Sci.*, 2015, **112**, 6277–6282.

9 H. Althaus, M. Chudacoff, R. Hischier, N. Jungbluth, M. Osses, A. Primas and S. Hellweg, *Life cycle inventories of chemicals. Ecoinvent report No.8, v2.0*, Dübendorf, Switzerland, 2007.

10 T. Ren, M. Patel and K. Blok, *Energy*, 2006, **31**, 425–451.
